# Deletion of *Toxoplasma* Rhoptry Protein 38 (PruΔ*rop38*) as a Vaccine Candidate for Toxoplasmosis in a Murine Model

**DOI:** 10.3390/biomedicines10061336

**Published:** 2022-06-06

**Authors:** Yayun Wu, Zihui Zhou, Zhu Ying, Ying Xu, Jing Liu, Qun Liu

**Affiliations:** 1 National Animal Protozoa Laboratory, College of Veterinary Medicine, China Agricultural University, Beijing 100193, China; yayunwu@cau.edu.cn (Y.W.); zzh_9833@163.com (Z.Z.); YZyingzhu@163.com (Z.Y.); xuying0516@mail.xjtu.edu.cn (Y.X.); liujingvet@cau.edu.cn (J.L.); 2 Key Laboratory of Animal Epidemiology of the Ministry of Agriculture, College of Veterinary Medicine, China Agricultural University, Beijing 100193, China

**Keywords:** toxoplasmosis, PruΔ*rop38*, pathogenicity, protective efficacy, mice

## Abstract

Toxoplasmosis is a serious zoonotic disease that threatens human and animal health. Here, we evaluated the vaccine potential of the deletion of *Toxoplasma* rhoptry protein 38 (PruΔ*rop38*) through its pathogenicity and immunoprotective efficacy in mice. Mice inoculated intraperitoneally with 1 × 10^3^, 2 × 10^3^, or 4 × 10^3^ PruΔ*rop38* showed no visible signs, whereas mice inoculated with 1 × 10^3^ parental Pru strain showed obvious wasting and bow-back, suggesting a significantly lower pathogenicity of PruΔ*rop38* in mice. Vaccination with 1 × 10^2^ PruΔ*rop38* triggered a mixed Th1/Th2 response (Th1 response predominant), with higher IgG, IgG2a, and IgG1 levels in serum from week 3 to week 12, and a significant increase in IFN-γ, IL-12, and IL-10 in suspensions of splenocytes at 30 or 60 days post-immunization. All vaccinated mice survived when infected intraperitoneally with tachyzoites (RH, Pru, VEG, or TgcatBJ1) or when infected orally with cysts (Pru or ME49). The brain parasite burden during Pru tachyzoite, Pru cyst and ME49 cyst challenges were significantly reduced in vaccinated mice. The duration of immunization showed that vaccination with PruΔ*rop38* could protect mice from challenge with different varied genotypes of *Toxoplasma* strains against different routes of infection. Collectively, these findings indicate that PruΔ*rop38* is an attenuated strain that provides long-term protective efficacy against acute or chronic toxoplasmosis in mice.

## 1. Introduction

*Toxoplasma gondii* is an obligate intracellular protozoan with a wide host range that can infect almost all warm-blooded animals and is capable of causing toxoplasmosis in both humans and animals [1,2]. Toxoplasmosis remains one of the world’s leading causes of illness and death, resulting in more than 200 million clinical cases of human zoonotic infections each year, which develop as fatal encephalitis or pneumonia in immunodeficient patients undergoing AIDS treatment [3]. Infected pregnant women may suffer a miscarriage or their newborn babies may suffer from congenital toxoplasmosis [4]. *Toxoplasma* infection is acquired by ingestion of water and food, contaminated with *T. gondii* oocysts or cysts. Ingestion of these life stages rapidly converts them into tachyzoites in the host for mass reproduction, leading to acute infection [5].

Pyrimethamine and sulfadiazine are commonly available treatments for the control of toxoplasmosis, while failure rates and severe side effects of the drugs remain significant, targeting only the active stage of infection [6]. Vaccines are expected to be the most promising method to prevent and cure toxoplasmosis [7]. The development of toxoplasmosis vaccines has evolved from traditional vaccines (e.g., live and inactivated vaccines) to molecular biotechnology vaccines (e.g., recombinant live vector vaccines, recombinant subunit vaccines, and DNA vaccines), but none of these have yet been able to eliminate the formation of cysts effectively. Although live-attenuated vaccines are assumed to induce a stronger immune response against infection and provide more stable protection for experimental animals [8], only the S48 live-attenuated strain (Toxovax) is a licensed vaccine. S48 is restricted to sheep to reduce abortion and congenital toxoplasmosis, which remains a risk for vulnerable hosts and provides partial protection for sheep [9]. The development of a safe and effective vaccine to prevent the transmission of toxoplasmosis is of great importance.

The progress in the development of live-attenuated vaccines has always been facilitated by CRISPR/Cas9-mediated homologous recombination [10], and several gene-deletion strains have been evaluated for their potential use in vaccine research against toxoplasmosis in mice [11,12,13,14]. *T. gondii* secretory proteins from three obligate secretory organs (rhoptries, micronemes and dense granules) have been shown to manipulate the host immune response [15]. We found that deletion of TgROP38 promoted production of pro-inflammatory cytokines (IL-18 secretion) in early infection with the Pru strain, and PruΔ*rop38* exhibited attenuated virulence in mice [16]. Another comparison of the roles of rhoptry proteins in establishing chronic infection indicated that TgROP38 is essential for the chronic stage, with severely reduced brain cyst burdens in PruΔ*rop38*/*29*/*19* [17] compared to the parental Pru strain. Considering that PruΔ*rop38* may have the potential to be a live-attenuated vaccine candidate for toxoplasmosis, we assessed the pathogenicity, immunogenicity and protective efficacy of PruΔ*rop38* in mice.

## 2. Materials and Methods

### 2.1. Animals and Ethical Approval

Female CD-1 mice (6 to 7 weeks) were purchased from Beijing Vital River Laboratory Animal Technology Co., Ltd. (Beijing, China, LICENCE No. SCXK (Jing) 2021-0006). Immunogenicity and protection protocols were in accordance with the recommendations of the Guide for the Care and Use of Laboratory Animals, Ministry of Science and Technology of China. The procedures were reviewed and approved by the Laboratory Animal Welfare and Animal Experimental Ethical Committee of China Agricultural University (Certificate No.: CAU-AW31901202-2-1).

### 2.2. Parasites

Tachyzoites of PruΔ*rop38*, *T*. *gondii* type I strain RH, type II strain Pru, type III strain VEG, and ToxoDB #9 strain TgCatBj1 [18] were propagated in human foreskin fibroblast cells (obtained from ATCC, Manassas, VA, USA), which were cultured in Dulbecco’s modified Eagle’s medium (DMEM, Macgene, CM17206, Beijing, China) supplemented with 2% fetal bovine serum (FBS, Gibco, Rockville, MD, USA) and incubated at 37 °C and 5% CO_2_.

Cysts of *T. gondii* type II Pru and ME49 strain were maintained in CD-1 mice by oral passaging of cysts in mice.

### 2.3. Virulence Comparison of PruΔrop38 and Pru Strain

To determine the virulence of PruΔ*rop38* in vivo, different doses of freshly egressed Pru (1 × 10^3^ and 2 × 10^3^) or PruΔ*rop38* (1 × 10^3^, 2 × 10^3^, 4 × 10^3^, and 8 × 10^3^) tachyzoites were injected intraperitoneally (i.p.) into mice (five mice per group), followed by daily monitoring of body weight gain status, clinical symptoms [19], and survival of the mice monitored for another 30 days. Pru strain is less virulent than genotype I strains; mice infected with 1 × 10^3^ Pru caused obvious clinical signs [16], so mice were infected with 1 × 10^3^, 2 × 10^3^, 4 × 10^3^, and 8 × 10^3^ PruΔ*rop38* to determine the degree of reduced pathogenicity. Initial screening at different doses can also provide an understanding of the safety range for vaccine strain.

### 2.4. Dynamic Distribution of PruΔrop38 In Vivo

To determine the dynamic distribution of immunized PruΔ*rop38* strains, five mice per group were infected by i.p. injections of 1 × 10^2^, 5 × 10^2^, and 1 × 10^3^ PruΔ*rop38* tachyzoites, respectively. Three mice from each group were selected for DNA extraction from spleen, kidney, liver, lung, heart, brain tissue, mesenteric lymph nodes and peritoneal fluid collection weekly after inoculation, in accordance with the instructions of Genomic DNA Extraction Kit (Aidlab Biotech, Beijing, China). To screen for *T. gondii*, amplification was performed using the 529 [20], ITS-1 [21] and B1 [22] genes, reaction conditions were 7 min incubation at 94 °C, followed by 35 cycles of 1 min at 94 °C, 1 min at 55 °C, 1 min at 72 °C and a final 10 min incubation at 72 °C as described previously (Supplementary Table S1).

### 2.5. Brain Parasite Burden Assay

Groups of 5 mice were infected by i.p. injection of 1 × 10^2^, 5 × 10^2^, and 1 × 10^3^ PruΔ*rop38* tachyzoites, and a group of 5 mice infected by i.p. injection of 1 × 10^3^ Pru were used as control. Thirty days post-infection, the brains of mice infected with Pru and PruΔ*rop38* strains were harvested and homogenized in 2 mL volumes of sterile PBS using homogenizers. Cyst counts were performed on 200 μL of brain homogenates by DBA-FITC (Vector Laboratories, Burlingame, CA, USA) staining [23]. Cyst images were observed using a fluorescence microscope (Olympus Co., Tokyo, Japan). To quantify the brain parasite burden of mice, the number of parasites and DNA concentration in each brain tissue sample were detected by calculating the 529 gene of *Toxoplasma* and the 28S rRNA gene of the brain, respectively. Reaction conditions were 7 min incubation at 94 °C, followed by 45 cycles of 1 min at 94 °C, 1 min at 60 °C, 1 min at 72 °C and a final 10 min incubation at 72 °C using quantitative real-time PCR with the SYBR Green Master Mix (Vazyme Biotech, Nanjing, China) [24] (Supplementary Table S1). The DNA concentration was measured by spectrophotometry. Data were analyzed by a Roche LightCycler 480 (Roche, Basel, Switzerland).

### 2.6. Immunization and Challenges

CD-1 mice were randomly divided into two equal groups, and vaccinated by i.p. inoculation with 1 × 10^2^ tachyzoites of PruΔ*rop38* or PBS.

To evaluate the protective efficacy against toxoplasmosis, 30 and 60 days after vaccination, mice were challenged with different genotypes of *T. gondii* strains (five mice per group), and mice were infected type I strain RH, type II strain Pru, type III strain VEG, and ToxoDB #9 strain TgCatBj1 tachyzoites by i.p. inoculation. To mimic the natural infection route, mice were also orally infected with Pru and ME49 tissue cysts. Four weeks after challenge, the number of brain cysts in mice challenged with Pru tachyzoites, Pru cysts and ME49 cysts was determined under microscopic examination.

### 2.7. Toxoplasma IgG Antibody Assay

Three mice from the vaccination and control groups were selected for sera extraction every week after inoculation, and the serum samples were stored at −20 °C until assayed by ELISA to measure the levels of *Toxoplasma*-specific IgG and its two subtypes IgG1 and IgG2a. Briefly, 96-well plates were coated with 100 μL of *T. gondii* soluble antigens from RH strain [25] (TSA; 5 µg/mL) diluted overnight at 4 °C in coating buffer, and the plates were washed three times with PBST and blocked with 5% skim milk at room temperature. After washing 3 times with PBST, 100 μL serum samples (diluted 1:50 with 5% skim milk) were added to the wells and incubated at 37 °C for 1 h. The plates were then washed 3 times with PBST and incubated with horseradish peroxidase (HRP)-conjugated goat anti-mouse IgG, IgG1, or IgG2a secondary antibodies (1:5000 dilutions, Proteintech Group Inc., Chicago, CA, USA) at 37 °C for 1 h. After washing 3 times with PBST, the immune complexes were revealed by incubation with TMB as substrate, and the results were measured at OD 450 nm using a microplate reader (Synergy H(1), Biotek, Winooski, VT, USA), and all assays were performed in triplicate.

### 2.8. Cytokine Production Assay

Mice spleens from the vaccination and control groups were collected to compare cytokine production of splenocytes. Briefly, spleens were aseptically removed from three mice 30 and 60 days post-immunization and pressed through a 70 μm cell strainer to remove tissue debris. Splenocytes were extracted and purified using Lymphocyte Separation Medium (Dakewe Biotech, Beijing, China). Purified splenocytes were plated in 96-well plates (3 × 10^5^ per well) and cultured in RPMI 1640 (Macgene, CM302001, Beijing, China) supplemented with 8% FBS. Finally, the suspensions were stimulated with TSA (final concentration of 50 μg/mL), Concanavalin A (Solarbio, C8110, Beijing, China) as a positive control (ConA; final concentration 5 µg/mL) or RPMI 1640 medium alone as a negative control at 37 °C and 5% CO_2_ environment. Culture supernatants were collected at different time points, where levels of interleukin (IL)-10, interferon gamma (IFN-γ), and IL-12 were detected at 72, 96, and 96 h, respectively [12], following the manufacturer’s instructions for commercial ELISA kits (SEA111Mu, SEA049Mu, and SEA056Mu; Cloud-Clone Corp, Wuhan, China).

### 2.9. Statistical Analysis

Graphs were created and statistical analyses were performed by Prism 8 (GraphPad, San Diego, CA, USA). Statistical analyses were performed using unpaired Student’s *t*-test or one-way ANOVA analysis. A *p* value < 0.05 was considered statistically significant, * *p* < 0.05, ** *p* < 0.01, and *** *p* < 0.001.

## 3. Results

### 3.1. PruΔrop38 Exhibits Significantly Reduced Pathogenicity in Mice

**Figure 1:**
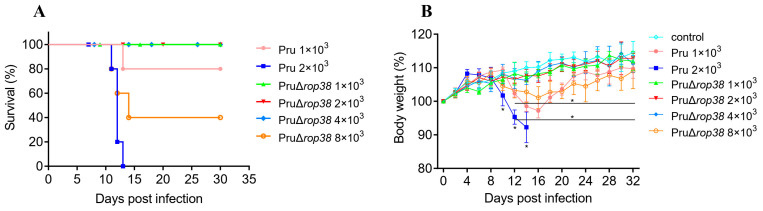
Pathogenicity of PruΔ*rop38* in mice. (**A**) Survival rate and (**B**) body weight gain of CD-1 mice infected with the parental Pru strain or the PruΔ*rop38* strain. Pru (1 × 10^3^ and 2 × 10^3^) or PruΔ*rop38* (1 × 10^3^, 2 × 10^3^, 4 × 10^3^, and 8 × 10^3^) tachyzoites were injected intraperitoneally (i.p.) into mice (*n* = 5), and daily monitoring of mice for 30 days (presented as the mean of body weight ± SD; * *p* < 0.05; one-way ANOVA with Tukey).

Mice inoculated intraperitoneally with the parental Pru strain showed varying degrees of symptoms, mice inoculated at a dose of 1 × 10^3^ showed obvious wasting and bow-back, with a survival rate of 80%, and mice inoculated at a dose of 2 × 10^3^ showed obvious signs with the death of all mice (Figure 1A,B). However, mice inoculated with doses 1 × 10^3^, 2 × 10^3^, and 4 × 10^3^ of PruΔ*rop38* showed no obvious clinical symptoms. Mice showed obvious wasting after inoculation with PruΔ*rop38* at a dose of 8 × 10^3^, with a survival rate of 40%. Based on the survival status, clinical symptoms and body weight gain data, the virulence of PruΔ*rop38* in mice was significantly attenuated compared to the parental Pru strain, indicating its availability as a candidate strain for vaccine potential studies.

### 3.2. Dynamic Distribution of PruΔrop38 in Mice

**Figure 2:**
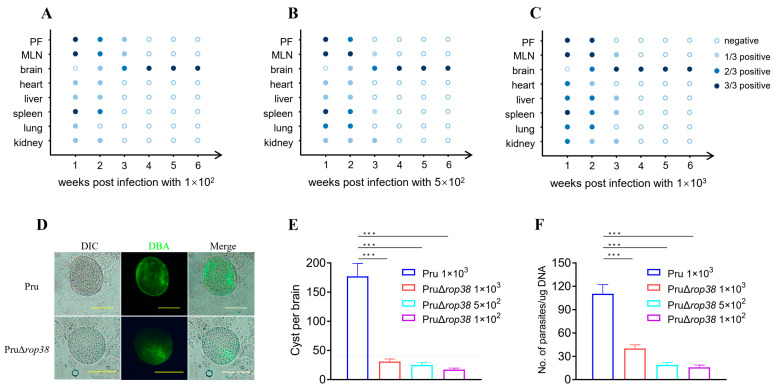
Dynamic distribution and effect on brain parasite burden of PruΔ*rop38* in mice. Amplification results of *T. gondii*-specific genes from organs and tissues in mice. Injections of (**A**) 1 × 10^2^, (**B**) 5 × 10^2^, and (**C**) 1 × 10^3^ PruΔ*rop38* tachyzoites, respectively. (**D**) Cyst counts were performed on brain homogenates by DBA-FITC staining, brain cyst from mice injected with PruΔ*rop38* and Pru were observed using a fluorescence microscope. (**E**) Immunofluorescence assays determined the number of brain cysts of Pru and PruΔ*rop38*. Pru (1 × 10^3^) or PruΔ*rop38* (1 × 10^2^, 5 × 10^2^, and 1 × 10^3^) tachyzoites were injected intraperitoneally (i.p.) into mice (*n* = 5) for 30 days. (Cyst walls of *T. gondii* were labeled with DBA-FITC (green), Scale bar = 50 µm). (**F**) The number of parasites and DNA concentration in each brain tissue were detected by calculating the 529 gene of *Toxoplasma* and the 28S rRNA gene of the brain with quantitative real-time PCR, the load of parasites = number of parasites/DNA concentration of brain tissue (presented as the mean ± SD; *** *p* < 0.001; one-way ANOVA with Tukey).

To further assess the pathogenicity in mice, we first observed the dynamic distribution and histological changes in mice inoculated intraperitoneally with PruΔ*rop38* at doses of 1 × 10^2^, 5 × 10^2^, and 1 × 10^3^. The amplification results showed that PruΔ*rop38* was widely distributed in all organ tissues except the brain in the first week after inoculation, and PruΔ*rop38* was also detected in the brain from the second week. The third week results revealed that *T. gondii*-specific genes were detected only in the peritoneal fluid, Mesenteric lymph nodes (MLN), and brain tissues with 1 × 10^2^ inoculation (Figure 2A), and in the MLN, brain, spleen, and kidney with 5 × 10^2^ inoculation (Figure 2B), as well as in the MLN, brain, liver, spleen, and kidney with 1 × 10^3^ inoculation (Figure 2C), however, no *T. gondii*-specific genes were detected from organs in three groups of organs from week 4 onwards, but rather from brain tissue. There was some degree of enlargement in the spleen and MLN without pathological changes, and no obvious histological lesions were seen on observation of the remaining organ and tissues. The dynamic distribution of PruΔ*rop38* in mice and histological observations further indicate that PruΔ*rop38* significantly reduces pathogenicity in mice.

### 3.3. Deletion of TgROP38 Significantly Reduces Brain Parasite Burden

To determine whether deletion of TgROP38 affects chronic infection in vivo at 30 days post-infection, we enumerated brain cysts in mice inoculated with 1 × 10^2^, 5 × 10^2^, and 1 × 10^3^ of PruΔ*rop38*, and we also infected CD-1 mice with Pru stain at a dose of 1 × 10^3^ as a control (Figure 2D). The average number of brain cysts in mice inoculated with Pru was 177, while the average number of brain cysts in mice inoculated with 1 × 10^2^, 5 × 10^2^ and 1 × 10^3^ of PruΔ*rop38* was 17 (*p* < 0.001), 25 (*p* < 0.001) and 31 (*p* < 0.001), respectively (Figure 2E). The parasite load in the brains of mice inoculated with 1 × 10^2^, 5 × 10^2^, and 1 × 10^3^ of PruΔ*rop38* was 15.84, 19.22, and 25.94 parasites per μg of brain DNA, respectively (Figure 2F), being significantly lower than that of Pru-inoculated mice (110.47 parasites per μg of brain DNA; *p* < 0.001). These results suggest that PruΔ*rop38* significantly reduces the brain parasite burden compared to the parental Pru strain.

### 3.4. Humoral Immune Responses Induced by PruΔrop38

**Figure 3:**
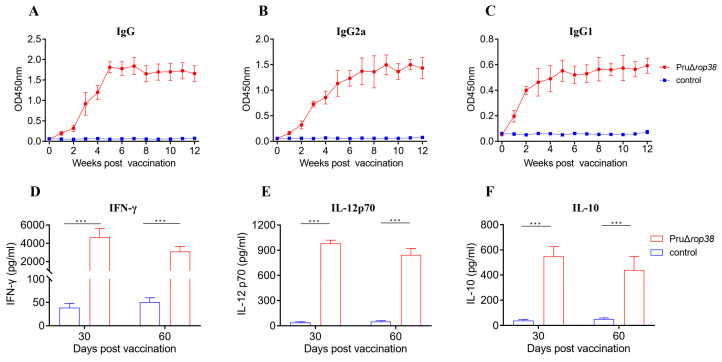
Humoral and cellular immune responses induced by PruΔ*rop38*. Levels of IgG and its subtype (IgG2a and IgG1) antibodies in the serum obtained from vaccinated and control mice. (**A**) IgG, (**B**) IgG2a, and (**C**) IgG1, respectively (*n* = 3; weekly serum collection after immunization). The levels of cytokines in the suspension of splenocyte in vaccinated and control mice. Splenocytes were collected at 30 or 60 days post-vaccination and co-incubated with TSA (50 μg/mL), and the levels of (**D**) IFN-γ, (**E**) IL-12, and (**F**) IL-10 were analyzed by ELISA (*n* = 3; presented as the mean ± SD; *** *p* < 0.001; Student’s *t*-test).

We next evaluated the potential of PruΔ*rop38* as an effective vaccine. To determine the humoral immune responses derived from PruΔ*rop38* immunization, we measured the antibody levels of specific anti-*T. gondii* IgG and IgG isotypes in the mouse serum. The safe immunization dose chosen was 1 × 10^2^ tachyzoites by i.p. inoculation from the immunoprotective efficacy comparison of mice from four immunization doses in mice (Supplementary Figure S1a–d). With weekly serum collection after immunization, ELISA results showed a continuous increase in the level of IgG antibodies from the first to the third week, and revealed significantly high levels from the third to the twelfth week after vaccination (Figure 3A).

To determine whether a Th1 and/or a Th2 humoral response was induced by vaccination with PruΔ*rop38*, sera from unvaccinated mice were also used as controls, and serum levels of both IgG2a (Figure 3B) and IgG1(Figure 3C) increased significantly from the second week onwards. In addition, the IgG1/IgG2a ratio was less than 1, demonstrating that PruΔ*rop38* induced a combined Th1/Th2 immune response after vaccination, with a predominant Th1 response.

### 3.5. Cellular Immune Responses Induced by PruΔrop38

Splenocytes harvested from vaccinated or control mice at 30 or 60 days post-immunization were stimulated with TSA, ConA or RPMI 1640 medium. The suspensions (stimulated with TSA) were measured by ELISA to assess the immunological memory and cellular immune responses induced by PruΔ*rop38*. The levels of IFN-γ (Figure 3D), IL-12 (Figure 3E) and IL-10 (Figure 3F) were markedly increased in immunized mice compared to those in control mice (*p* < 0.001) at both time points, indicating that immunizing mice with PruΔ*rop38* induces a mixed Th1/Th2 response and that Th1-type cell-mediated immune responses trigger leading protection against challenge.

### 3.6. Protection against Varied Genotypes of Tachyzoites

**Figure 4:**
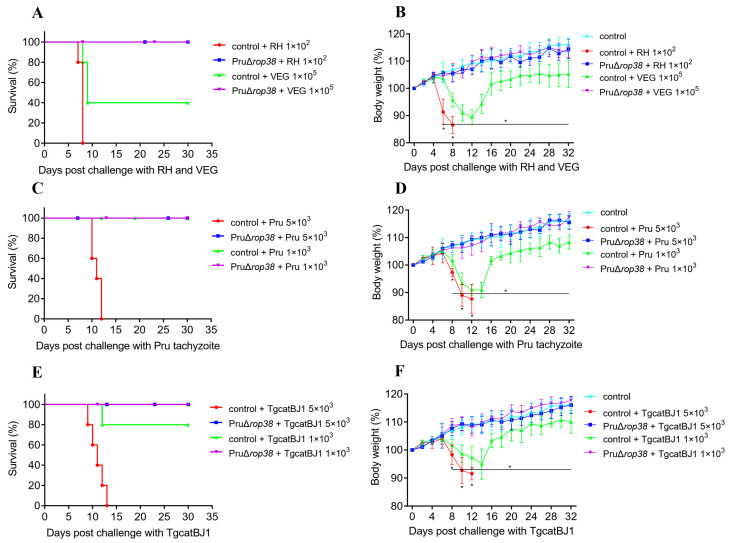
Protective efficacy against varied genotypes of tachyzoite challenge. Survival rate and body weight gain of vaccinated and control mice re-challenged with (**A**,**B**) RH (1 × 10^2^) and VEG (1 × 10^5^) tachyzoites, (**C**,**D**) Pru (1 × 10^3^ and 5 × 10^3^) tachyzoites, and (**E**,**F**) TgCatBj1 (1 × 10^3^ and 5 × 10^3^) tachyzoites, respectively. (*n* = 5; mice were injected intraperitoneally (i.p.); daily monitoring of mice for 30 days; presented as the mean of body weight ± SD; * *p* < 0.05; one-way ANOVA with Tukey).

To evaluate the protective efficacy against toxoplasmosis in mice, varied genotypes of tachyzoites (RH, Pru, VEG, and TgCatBj1) were used to infect mice intraperitoneally at corresponding doses. Control mice, challenged with lethal doses of 1 × 10^2^ RH, 1 × 10^5^ VEG, 5 × 10^3^ Pru, or 5 × 10^3^ TgCatBj1 tachyzoites, exhibited significant weight loss and bow-back, and all mice died within the second week after challenge, with only 40% of mice surviving the 1 × 10^5^ VEG infection (Figure 4A,B). Besides, control mice infected with 1 × 10^3^ Pru (Figure 4C,D) or 1 × 10^3^ TgCatBj1 (Figure 4E,F) tachyzoites (non-lethal dose) revealed continued weight loss at 8–14 days post-challenge, with body weight dropping to a minimum of 90.93% (*p* < 0.001) and 94.53% (*p* < 0.001) of their initial weight, respectively, and only one mice died with 1 × 10^3^ TgCatBj1. In contrast, vaccinated mice challenged with the four genotypes of tachyzoites showed normal weight gain (*p* > 0.05) and the same mental status as blank control mice. Taken together, PruΔ*rop38* may provide efficient protective efficacy against varied genotypes of RH, Pru, VEG, and TgCatBj1 tachyzoites challenge in mice.

### 3.7. Protection against Pru and ME49 Cyst

**Figure 5:**
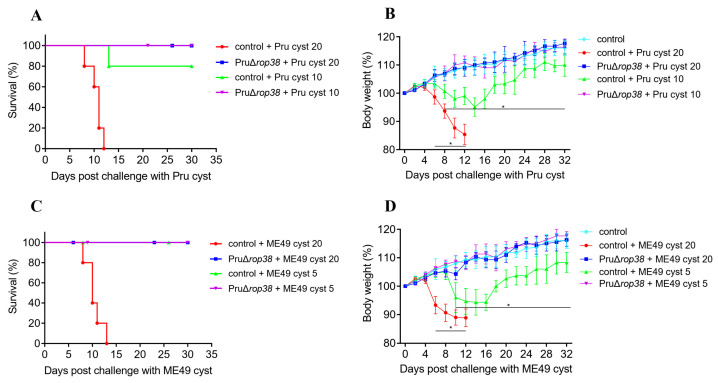
Protective efficacy against varied genotypes of cyst challenge. Survival rate and body weight gain of vaccinated and control mice re-challenged with (**A**,**B**) Pru (20 and 10) cysts and (**C**,**D**) ME49 (20 and 5) cysts (*n* = 5; mice were injected orally; daily monitoring of mice for 30 days; presented as the mean of body weight ± SD; * *p* < 0.05; one-way ANOVA with Tukey).

We also evaluated the protective efficacy of PruΔ*rop38* upon oral re-challenge with cysts, to simulate the natural transmission route; two groups of mice were infected with two doses of Pru and ME49 cysts, respectively. During the 30-day observation of survival, body weight and clinical signs, all control mice died within 8–13 days of infection with lethal doses of 20 Pru (Figure 5A,B) or ME49 cysts (Figure 5C,D), with a more severe weight loss (*p* < 0.001). Mice were also infected with 10 Pru or 5 ME49 cysts (pathogenic dose) to assess the protective efficacy against chronic infection, and all control mice survived the observation period while their body weight dropped to a minimum of 95.17% (*p* < 0.001) (Figure 5B) and 94.36% (*p* < 0.001) (Figure 5D) of their initial weight, respectively. Instead, all vaccinated mice challenged with Pru or ME49 cysts survived, and no obvious clinical signs were observed, and none of the weight gains were significant compared to blank control mice (*p* > 0.05). Taken together, PruΔ*rop38* may also provide efficient protective efficacy in mice that survive during acute or chronic infection with Pru and ME49 cysts.

### 3.8. Significant Reduction in Brain Parasite Burden in Challenging Type II Strains

**Figure 6:**
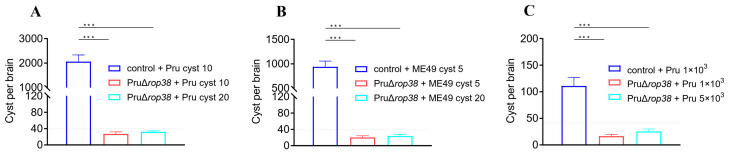
Effect on brain parasite burden when challenged with *T. gondii* type II strains. The brain from vaccinated and control mice were harvested after challenge with 30 days, and cysts were observed by IFA and then the number of cysts were determined by DBA-FITC staining. (**A**) Pru cyst challenge (20 and 10), (**B**) ME49 cyst challenge (20 and 5), and (**C**) Pru tachyzoite (5 × 10^3^ and 1 × 10^3^) challenge, respectively. (*n* = 5; presented as the mean of cyst number ± SD; *** *p* < 0.001; one-way ANOVA with Tukey).

Another aspect of evaluation of the protective efficacy against chronic infection is the brain parasite burden, so we counted the number of brain cysts in mice challenged with type II strains. The average number of cysts in control mice challenged with 10 Pru cysts, 5 ME49 cysts, and 1 × 10^3^ Pru tachyzoites was 2062, 940, and 111, respectively (Figure 6A–C). However, the number of cysts in vaccinated mice was about 27 (*p* < 0.001), 20 (*p* < 0.001), and 17 (*p* < 0.001), respectively. Additionally, the number of cysts in vaccinated mice challenged with 20 Pru cysts, 20 ME49 cysts, and 5 × 10^3^ Pru tachyzoites (lethal doses in control mice) was 32 (*p* < 0.001), 24 (*p* < 0.001), and 25 (*p* < 0.001), respectively. Vaccination with PruΔ*rop38* has the ability to significantly reduce the brain parasite burden of the challenging type II strains and provide protection against *T. gondii* chronic infection.

### 3.9. Vaccination with PruΔrop38 Provides Long-Time Protection in Mice

**Figure 7:**
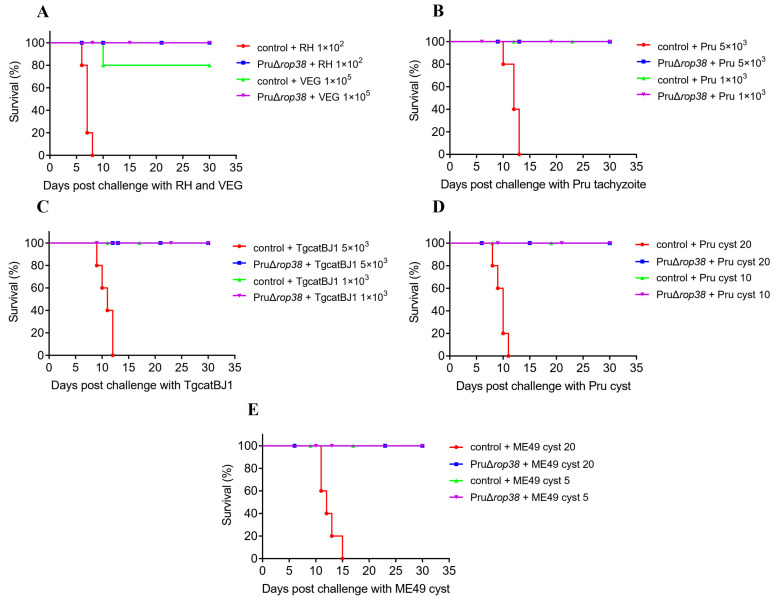
The duration of immunity with PruΔ*rop38*. Survival rate of vaccinated and control mice re-challenged with (**A**) RH (1 × 10^2^) and VEG (1 × 10^5^) tachyzoites, (**B**) Pru (1 × 10^3^ and 5 × 10^3^) tachyzoites, and (**C**) TgCatBj1 (1 × 10^3^ and 5 × 10^3^) tachyzoites, (**D**) Pru (20 and 10) cysts, and (**E**) ME49 (20 and 5) cysts, respectively (*n* = 5; mice were injected with tachyzoites intraperitoneally (i.p.) or with cysts orally; daily monitoring of mice for 30 days).

To evaluate the duration of immunity with PruΔ*rop38*, we finally assessed whether mice were able to resist *T. gondii* challenge 60 days after vaccination with PruΔ*rop38*. When challenged with lethal doses of tachyzoites (1 × 10^2^ RH, 1 × 10^5^ VEG, 5 × 10^3^ Pru, or 5 × 10^3^ TgCatBj1) or cysts (20 Pru or 20 ME49), control mice exhibited serious clinical signs (wasting, bow-back and depression), and all mice died at the second week (Supplementary Figure S2a–e), despite all vaccinated mice surviving challenges with varied genotypes of *T. gondii* strains (Figure 7A–E). When challenged with pathogenic doses of tachyzoites (1 × 10^3^ Pru, or 1 × 10^3^ TgCatBj1) or cysts (10 Pru or 5 ME49), all control mice survived (Figure 7B–E), but body weight dropped to 92.96% (*p* < 0.001), 93.16% (*p* < 0.001), 94.33% (*p* < 0.001), and 92.61% (*p* < 0.001) of the initial weight, respectively (Supplementary Figure S2b–e). Similarly, all vaccinated mice exhibited relatively stable weight and with no obvious clinical signs.

The average number of cysts in control mice infected with 1 × 10^3^ Pru tachyzoites, 10 Pru cysts and 5 ME49 cysts was 1690, 850, and 157, respectively (Supplementary Figure S3a–c). However, the number of cysts in vaccinated mice was about 29 (*p* < 0.001) and 35 (*p* < 0.001), and 24 (*p* < 0.001), respectively. In addition, the number of cysts in vaccinated mice challenged with 20 Pru cysts, 20 ME49 cysts, and 5 × 10^3^ Pru tachyzoites (lethal doses in control mice) was 31 (*p* < 0.001), 36 (*p* < 0.001), and 26 (*p* < 0.001), respectively. Taken together, vaccination with PruΔ*rop38* remains able to significantly reduce the brain parasite burden of the challenging type II strains, providing long-term efficacy against acute or chronic toxoplasmosis in mice.

## 4. Discussion

Toxoplasmosis is a serious zoonotic disease that threatens human and animal health, and vaccination strategies are considered ideal to prevent infection. The objectives are to prevent congenital toxoplasmosis in both women and livestock, prevent/reduce *T. gondii* tissue cysts in food-producing animals and oocyst shedding in cats [8]. While various approaches have been attempted to develop and achieve effective anti-*T. gondii* vaccines with varying degrees of protective immunity, the field of live-attenuated vaccine development has been carried out for safer vaccines for advantages in terms of protective efficacy [7]. Due to the essential roles of TgROP38 in the secretion of pro-inflammatory cytokines [16] and the attenuated virulence and reduced cyst burdens exhibited by PruΔ*rop38* in mice [17], the objective of this study was to evaluate the potential PruΔ*rop38* as an applicable vaccine candidate and assess its pathogenicity, immunogenicity and protective efficacy.

Survival status was the primary factor determining the pathogenicity of PruΔ*rop38* in vivo, besides, we also focused on clinical symptoms and body weight gain of mice after inoculation with different doses of Pru (1 × 10^3^ and 2 × 10^3^) or PruΔ*rop38* (1 × 10^3^, 2 × 10^3^, 4 × 10^3^, and 8 × 10^3^) tachyzoites. No obvious signs (wasting and bow-back) or deaths were seen even when inoculated with 4 × 10^3^ PruΔ*rop38*, demonstrating the attenuated pathogenicity of the candidate to mice. *T. gondii*-specific genes were only detected in the brain from week 4 onwards, and deletion of TgROP38 significantly reduced the brain parasite burden compared to the parental Pru strain. These data indicate that PruΔ*rop38* conforms to a number of characteristics that make it an ideal live-attenuated vaccine. Pathological changes in organs and tissues were also observed, with some degree of enlargement in the spleen and MLN, and no obvious histological lesions observed in the remaining organ and tissue. The final vaccination dose (1 × 10^2^ per mouse) was considered to significantly reduce pathogenicity and provide high biosafety in mice.

As with live-attenuated vaccine candidates [12], immunization with PruΔ*rop38* induces strong humoral and cellular immune responses. The level of specific anti-*T. gondii* IgG and IgG two isotypes (IgG1 and IgG2a) revealed a significantly high level from week 3 to week 12 after vaccination, since the IgG1/IgG2a ratio was less than 1, PruΔ*rop38* induces a combined Th1/Th2 immune response, in which the Th1 response predominates. In contrast, IgG activated by B-cell response can regulate immune cells and complement to inhibit the infective capacity of *T. gondii* [26].

At 30 or 60 days post-vaccination, the secretion of IFN-γ, IL-12 and IL-10 in the immunized mice splenocytes significantly increased under the TSA stimulation, indicating a Th1-type cell-mediated immune response was induced predominantly. IL-12 and IFN-γ trigger multiple intracellular responses to clear *T. gondii* by activating cytotoxic lymphocytes and natural killer cells, leading to protection against acute *T. gondii* infection [27,28]. However, excessive pro-inflammatory cytokine (IFN-γ and IL-12) responses usually lead to death in mice during acute infection, and as an anti-inflammatory cytokine, IL-10 can downregulate the expression of IFN-γ and IL-12, which in turn reduces the inflammatory response [29]. Immunogenicity assessment showed that vaccination with PruΔ*rop38* rapidly activated humoral and cellular immune responses to specifically recognize the *Toxoplasma* antigen.

PruΔ*rop38* provides long-term efficacy against acute and chronic toxoplasmosis in mice. For *Toxoplasma* it is now apparent that, besides the three typical genotypes (type I, type II and type III) described worldwide, there exist many atypical *T. gondii* genotypes, with a high degree of genetic diversity [30,31]. As a result, we used varied genotypes of tachyzoites (RH, Pru, VEG, and TgcatBJ1) to assess the protective efficacy of PruΔ*rop38*, with TgcatBJ1 being a ToxoDB#9 genotype strain isolated from stray cats [18]. Several atypical isolates are also commonly used in experiments to evaluate the efficacy of vaccine strains, such as the ToxoDB#9 genotype tachyzoites PYS [7], TgC7 [32], and C7719 [13], as well as the ToxoDB#3 tachyzoites WH-1 [33]. PruΔ*rop38* could provide effective protection to mice against challenge by tachyzoites of varied genotypes. However, intraperitoneal injection does not represent the natural route of infection, so Pru and ME49 cysts were orally challenged to objectively assess the protective efficacy, and PruΔ*rop38* could provide efficient efficacy in mice that survived during acute or chronic infection with Pru and ME49 cysts. Additionally, vaccination with PruΔ*rop38* was able to significantly reduce the brain parasite burden of the challenging type II strains in chronic infections. Taken together, PruΔ*rop38* is a potential live-attenuated vaccine candidate. However, the current study is limited to the mouse model, so more studies are needed to evaluate the effectiveness and safety of the vaccine candidate for food-producing animals or cats.

## 5. Conclusions

In summary, virulence, dynamic distribution, and brain parasite burden assays indicated that PruΔ*rop38* was significantly less pathogenic in mice compared to the parental Pru strain. Vaccination with 1 × 10^2^ PruΔ*rop38* triggered high levels of serum IgG, IgG2a, and IgG1 from week 3 to week 12, and a significant increase in IFN-γ, IL-12, and IL-10 in splenocyte suspensions at 30 or 60 days post-immunization. PruΔ*rop38* provides long-term efficacy against acute and chronic toxoplasmosis in mice and significantly reduces the brain parasite burden of the challenging *T. gondii* type II strains.

## Data Availability

Not applicable.

## Supplementary Materials

The following supporting information can be downloaded at: https://www.mdpi.com/article/10.3390/biomedicines10061336/s1. Figure S1: Protective efficacy comparison of four doses of vaccination; Figure S2: Body weight of mice in evaluation of duration of immunity with PruΔ*rop38*; Figure S3: Effect on brain parasite burden in the evaluation of duration of immunity with PruΔ*rop38*; Table S1: The primers used in this study.
