# Supplementary material for: Deletion of Toxoplasma Rhoptry Protein 38 (PruΔrop38) as a Vaccine Candidate for Toxoplasmosis in a Murine Model
Source: Biomedicines. 2022 Jun 6;10(6):1336. doi: 10.3390/biomedicines10061336 (PMC9220005; doi:10.3390/biomedicines10061336)
Supplement: Supplementary file 1 [file biomedicines-10-01336-s001.zip › Supplementary Material.pdf]

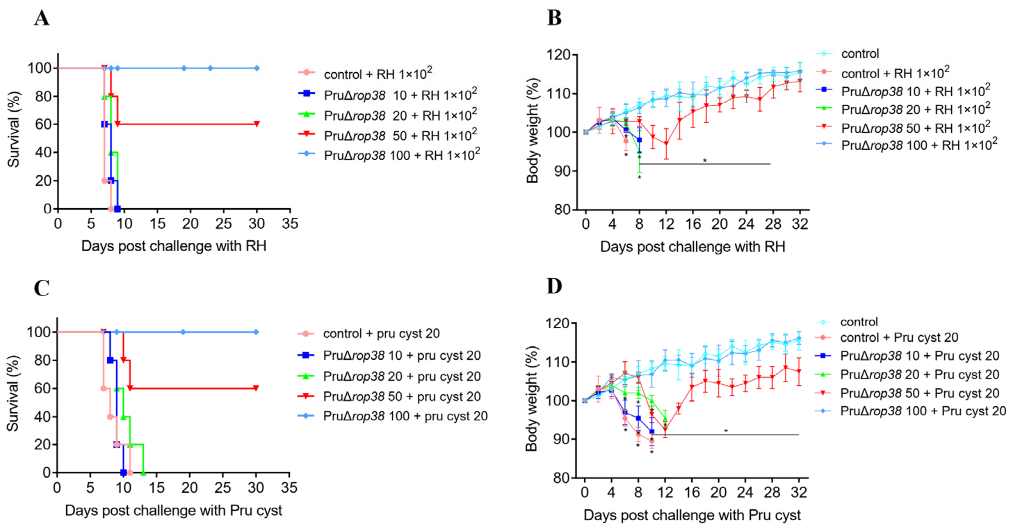

Figure S1. Protective efficacy comparison of four doses of vaccination. Survival rate and body weight gain of mice re-challenged with 1×10^{2} RH tachyzoites(**A,B**) or with 20 Pru cysts (**C,D**; lethal dose in mice). Mice were inoculated at four PruΔ*rop38* immunization dose (10, 20, 50, and 100), thirty days post vaccination, vaccinated and control mice were re-challenged with RH or Pru cyst (n = 5; daily monitoring of mice for 30 days; presented as the mean of body weight ± SD; * $p < 0.05$; One-way ANOVA with Tukey).

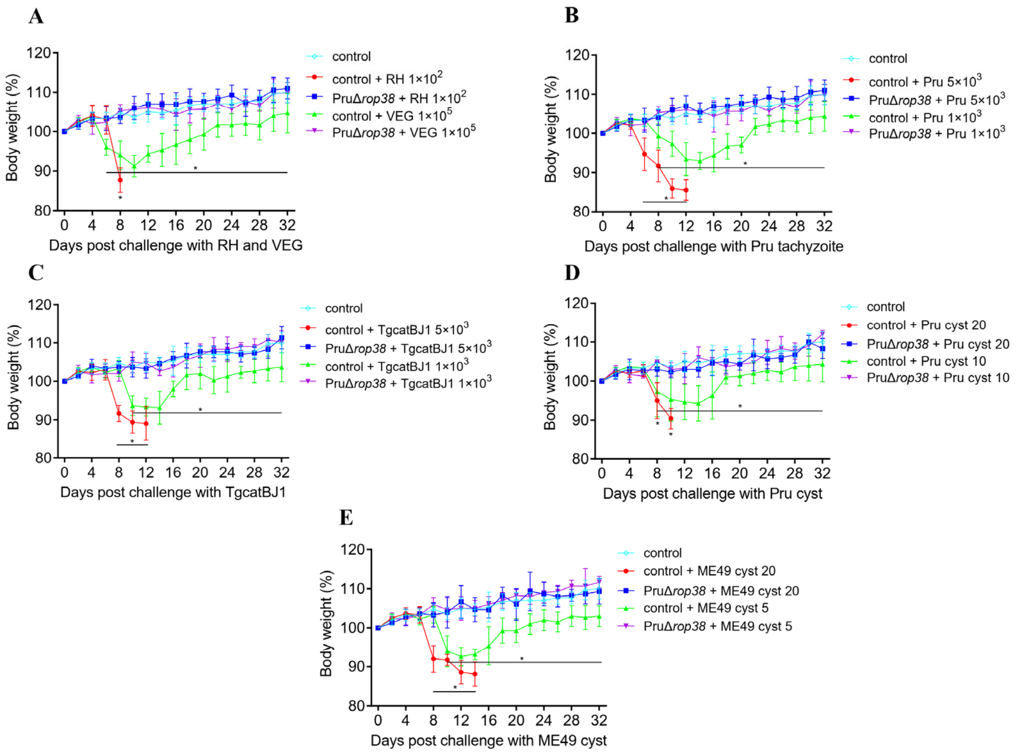

Figure S2. Body weight of mice in evaluation of duration of immunity with PruΔ*rop38*. body

weight gain of vaccinated and control mice re-challenged with (**A**) RH (1×10^2) and VEG (1×10^5) tachyzoites, (**B**) Pru (1×10^3 and 5×10^3) tachyzoites, and (**C**) TgCatBj1 (1×10^3 and 5×10^3) tachyzoites, (**D**) Pru (20 and 10) cysts, and (**E**) ME49 (20 and 5) cysts, respectively (n = 5; mice were injected with tachyzoites intraperitoneally (i.p.) or with cysts orally; daily monitoring of mice for 30 days; presented as the mean of body weight ± SD; * $p < 0.05$; One-way ANOVA with Tukey).

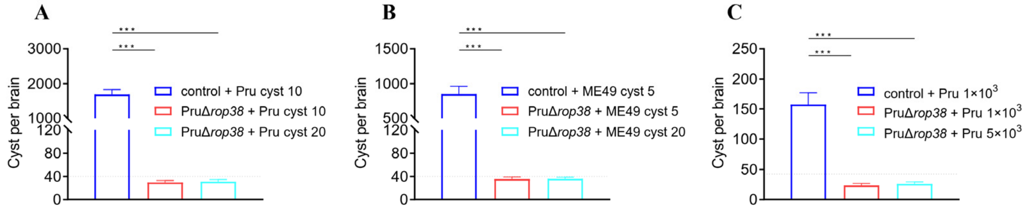

Figure S3. Effect on brain parasite burden in the evaluation of duration of immunity with PruΔ*rop38*. The brain from vaccinated and control mice were harvested after challenge with 30 days, and cysts were observed by IFA and then the number of cysts were determined by DBA-FITC staining. (**A**) Pru cyst challenge (20 and 10), (**B**) ME49 cyst challenge (20 and 5), and (**C**) Pru tachyzoites (5×10^3 and 1×10^3) challenge, respectively. (n = 5; presented as the mean of cyst number ± SD; *** $p < 0.001$; One-way ANOVA with Tukey).

Table S1. The primers used in this study.

Locus	Sequence	Reference
529	CGCTGCAGGGAGGAAGACGAAAGTTG<br>CGCTGCAGACAGAGTGCATCTGGATT	20
B1	GGAACTGCATCCGTTCATGAG<br>TCTTTAAAGGGTTCGTGGTC<br>TGCATAGGTTGCAGTCACTG	22
ITS-1	TCAACCTTTGAATCCAAA<br>CGAGCCAAGACATCCATT<br>GTGATAGTATCGAAAGGTAT<br>ACTCTCTCTCAAATGTTCCT	21
28S	TGCCATGGTAATCCTGCTCA<br>CCTCAGCCAAGCACATACACC	24
