# Supplementary figures and images for: Deletion of Toxoplasma Rhoptry Protein 38 (PruΔrop38) as a Vaccine Candidate for Toxoplasmosis in a Murine Model
Source: Biomedicines. 2022 Jun 6;10(6):1336. doi: 10.3390/biomedicines10061336 (PMC9220005; doi:10.3390/biomedicines10061336)

**A**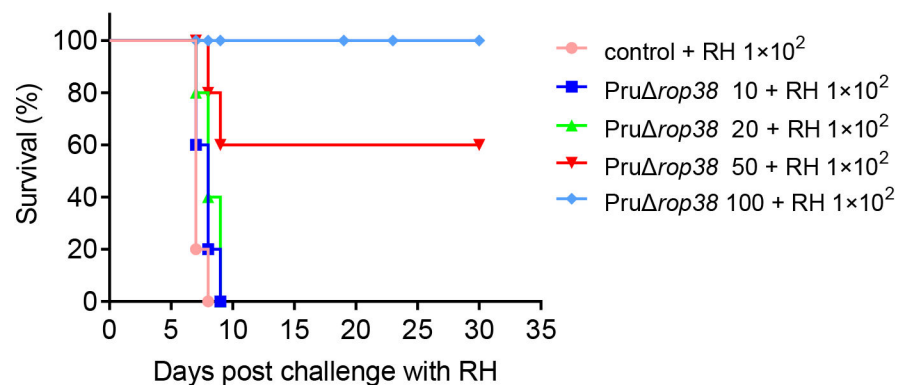**B**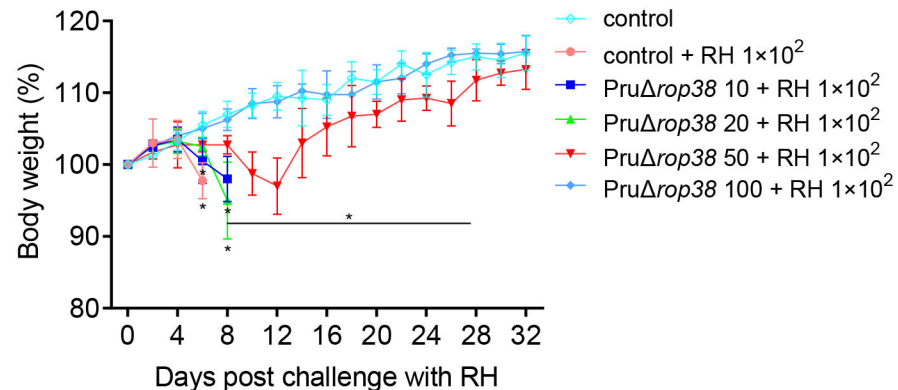**C**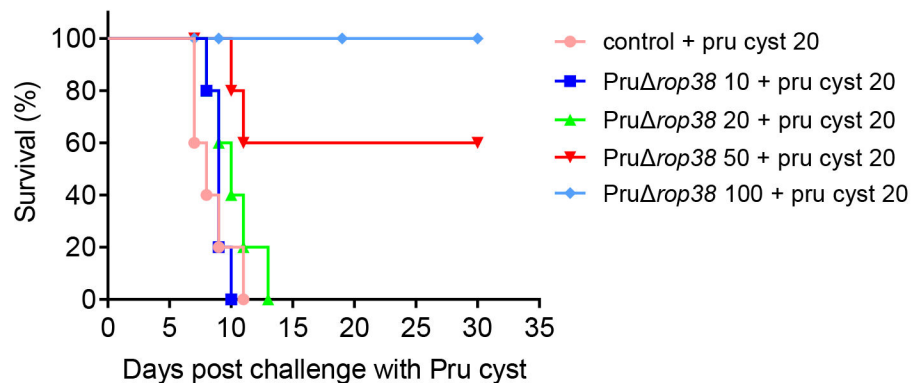**D**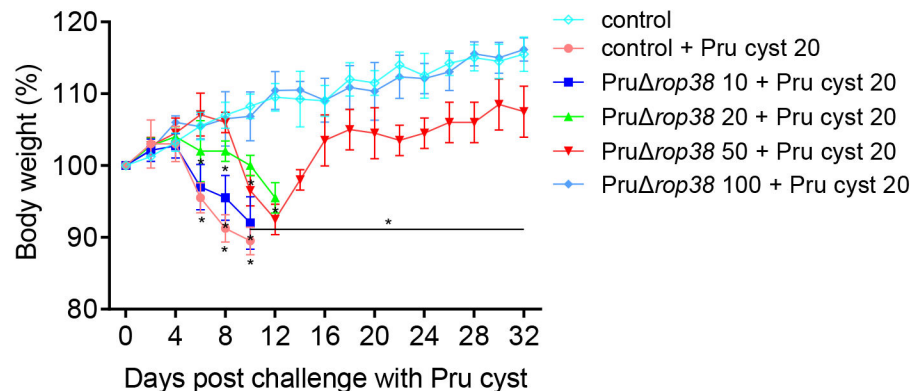

Supplement: Supplementary file 1 [file biomedicines-10-01336-s001.zip › Supplementary Figure S1.pdf]

**A**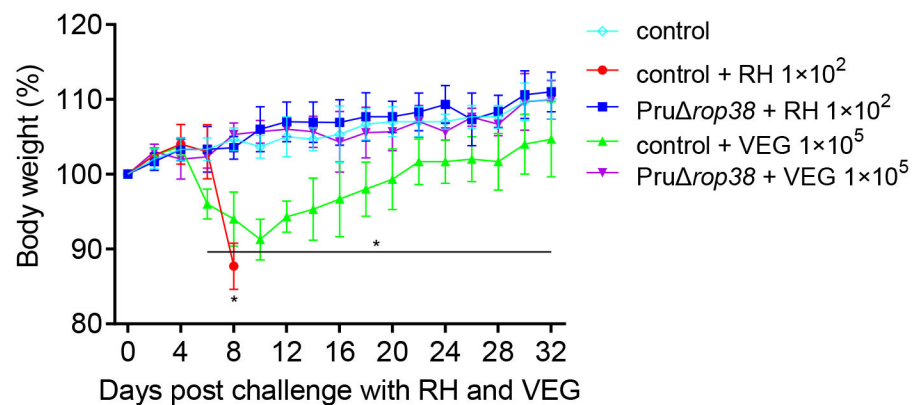**B**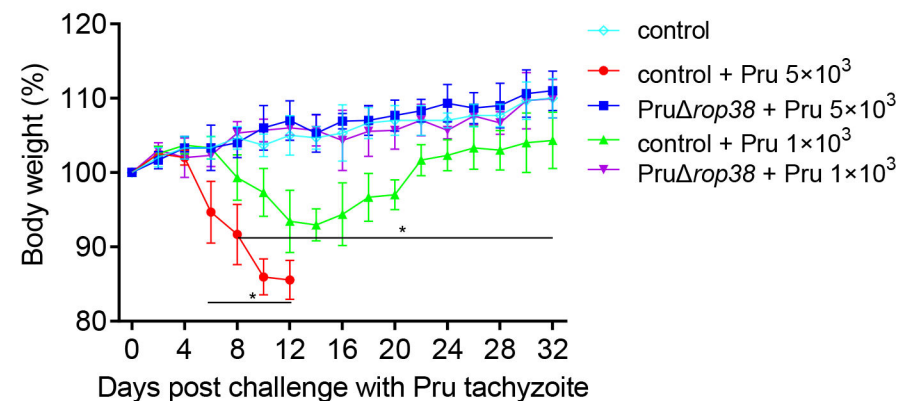**C**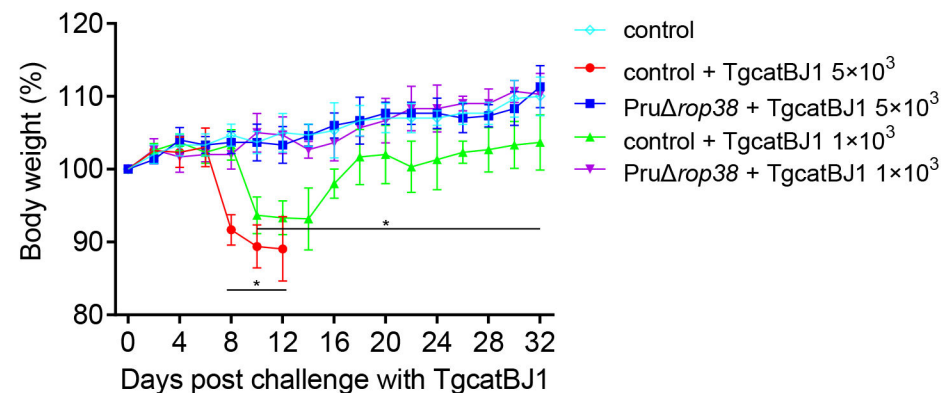**D**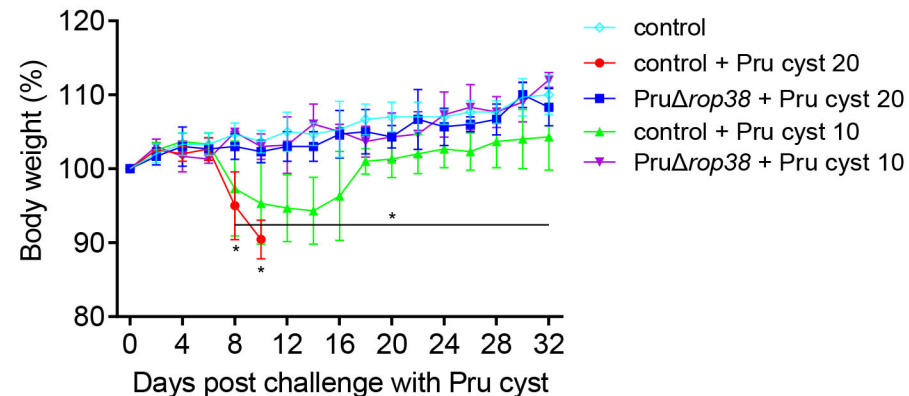**E**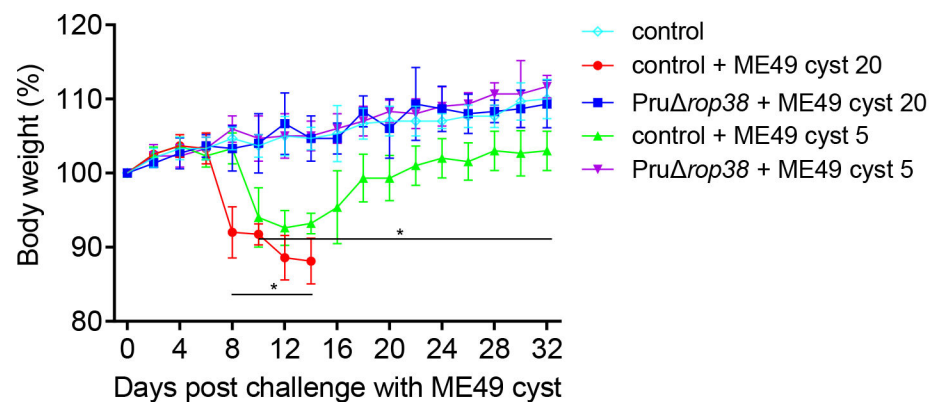

Supplement: Supplementary file 1 [file biomedicines-10-01336-s001.zip › Supplementary Figure S2.pdf]

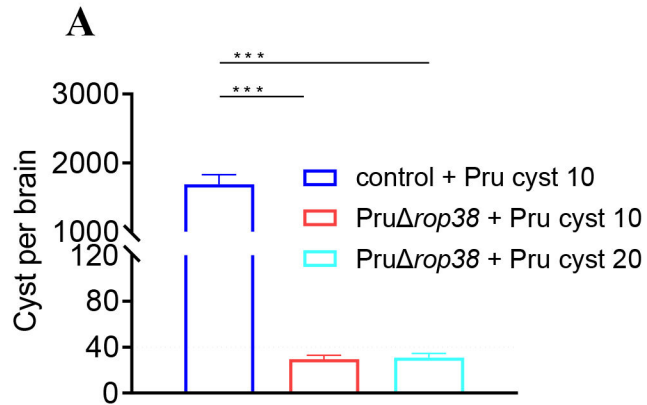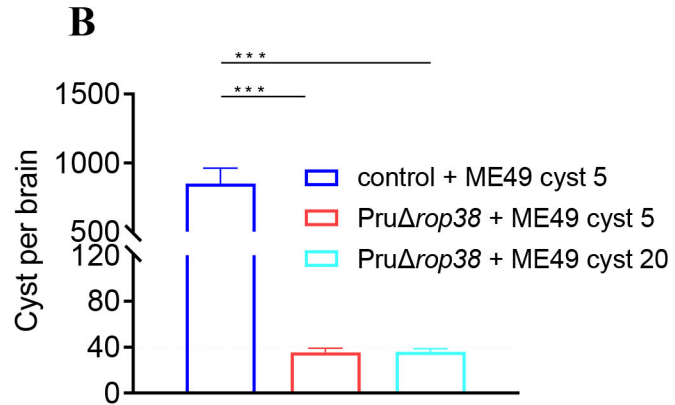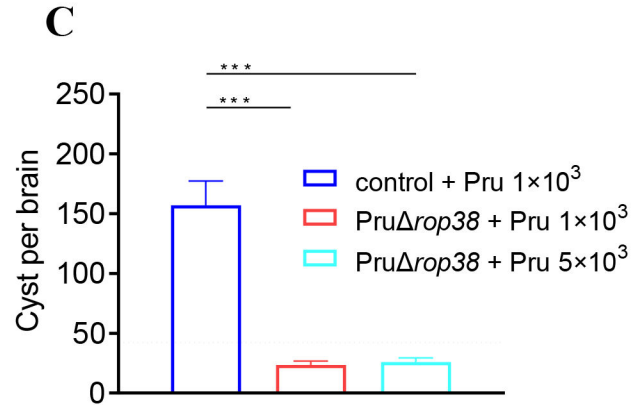

Supplement: Supplementary file 1 [file biomedicines-10-01336-s001.zip › Supplementary Figure S3.pdf]
